# Local and Constitutional Treatment of Diseases

**Published:** 1876-03

**Authors:** F. W. Sage


### LOCAL AND CONSTITUTIONAL TREATMENT OF DISEASES.

*Read Before the Ohio State Dental Society.*

BY F. W. SAGE, D. D. S.

"Medicine is justly distributed into *prophylactic*, or the art of preserving health;
And *therapeutic*, or the art of restoring it."—WATTS.

Whatever credit is due the dental profession for originality of aims and methods in the employment of medicines for the cure of diseases of the dental organs and associate parts; whatever the honors which accrue to members of the profession for discoveries of new properties in old and familiar remedies, for the modification of medicine in the treatment of lesions which come more especially under the observation of the oral surgeon, it must be acknowledged that to medical science and the medical profession we are beholden primarily for our knowledge of the remedies embraced in the dental pharmacopœia.

While this fact is indubitable it is as undeniably true that the medical profession is, in no slight degree, indebted to the dental profession, and to the many earnest scientists who are numbered in its ranks, and whose researches have served to develop dental science, for a more explicit knowledge of pathology, ætiology, and symptomatology, as referred to diseases of the oral cavity.

It would, perhaps, be impracticable, even if it were for any reason desirable, to show to just what extent the elder profession is indebted to the younger for the knowledge which has been supplied of the various specific disorders of the mouth; such as the anomalies of dentition, necrosis, ranula, diseases of the antrum, neuralgia, etc.; and for a better understanding of the means to be employed to effect a cure.

We may notice in this condition that to the dental profession is awarded the honor of the discovery of anæsthesia, and it is not perhaps, claiming too much to say that the dental profession has contributed most to a knowledge of the safest and most effective means of administering anæshetic agents. Be this as it may, the representatives of the two professions now draw upon the same common storehouse of nature for the material from which are derived medicinal remedies, and are mutually interested in the results of investigations and experiments which, in the light of scientific research, are yearly becoming more and more important and interesting.

Inasmuch as materia medica furnishes so extensive a variety of remedies, of almost any given class, which might be named; remedies which, in many instances, differ only immaterially in their essential properties, it is to be recommended to the majority of dental practioners that they confine themselves to the use of the least possible number available remedies of any one class. This course commends itself to our consideration for the reason that time and opportunity are not in many cases, at the command of the dental surgeon to observe very fully the action of medicines, and to intelligently comprehend their *rationale.* If we were inclined to modify this suggestion, in any respect, we should still utter our protest against the unreasonable practice, of which too many are guilty, of applying this, that and the other remedy to the correction of an abnormal development, without any intelligent apprehension of the requirements of the case or, indeed, of the influence of the remedy applied. But while we believe that the limiting of himself to the use of a few remedies, whose nature and operation he thoroughly understands, is the better and safer course for the practitioner of average or inferior scientific attainments. We would not be understood as offering any impediment to research; by competent men, in those fields where we have derived many invaluable additions to dental materia medica. So long as pathological conditions vary in their manifestations, and occasional idiosyncrasies operate to baffle and perplex the dentist, rendering futile his best directed and most intelligent efforts to effect a cure with the commonly prescribed remedies, so long will there be afforded encouragement to efforts which are directed to the discovery of the *active principle* of medicines and the elmination of crudities.

Medicine has never been an exact science, although it may be affirmed that we are tending more and more every year to specifics. The day has passed when the physician prescribed huge doses compounded of various and numerous ingredients, differing in their nature and influence, in the vague expectation of meeting the requirements of a case through the agency of some or other of those ingredients. So we as a profession have come to discard many remedies which were formerly in requisition, and have substituted others which are more definite in their action. Through a better understanding of pathology and ætiology we are able from the inspection of so delicate and minute an organ as the pulp of the tooth to discriminate between the various stages of disease, determining with reasonable exactness the degree of inflammation, congestion or suppuration which exists, and assigning the remedy accordingly.

In considering the subject of constitutional treatment, as the only means of cure in many cases of disease which now fall within the province of the dentist to treat, we are brought face to face with an important question, which the continual evolution of science persistently forces upon us. Never before to such an extent as at the present time, has the influence of constitutional disorders upon the merely local manifestation of disease, been appreciated. In our conventions and associations we have continually thrust upon our attention the importance of studying systematic conditions and of directing our efforts to effect a general renovation, and not merely a local amelioration of disorders. But just at this point we are confronted by the humiliating fact that with all the facilities which are afforded by our dental colleges, we cannot conscientiously claim to have attained to a degree of familiarity with the structure and functions of organs and of their relations to and dependence upon one another which enables us with any degree of confidence and certainty, to administer remedies for the correction of systemic disorders. We find ourselves honored by membership in a profession which already assumes the importance of a specialty of the medical profession, and that not by any intrusive effort, but nevertheless, irresistibly and inevitably by virtue of its inherent dignity and the constantly increasing scope of its ministrations to suffering humanity. How shall we keep pace with the requirements of our profession without a more liberal culture? How can we expect to ferret out and recognize systematic disorders and intelligently administer remedies, without a thorough medical education? Are we able to assign the causes of reflex action, or recognizing by the feeble light of science which now reaches us, the symptoms of constitutional derangements, can we with our present knowledge venture to trace them to their origin and remove what to us is now too often an insuperable impediment to the accomplishment of what should be infallible results? Must we always continue to deplore effects while we group in impenetrable darkness in a vain search after the cause? Surely since so much has been accomplished in the direction of the merely mechanical requirements of our profession, that it would seem that nothing remains to be accomplished, we shall not be content to wear the laurels of the artisan, while we acknowledge our incompetency to cope with local and constitutional affections through ignorance of a science upon which we must rest our claims, if at all, to recognition as a profession. In reviewing the progress of our profession during the last quarter of a century the most casual observer cannot fail to perceive from the tone of public sentiment what is expected of us in the near future. Nor is it difficult to predict what requisitions in another decade will be made on the dentist who attempts to mantain a claim to a position in the front ranks of the profession. Standing as we do on the very threshold of the medical profession, and appreciating as we do more fully than can those who make light of our aspirations, our need of a more liberal culture in order to prepare us for an intelligent investigation of morbid phenomena which in the progress of events have come for treatment within the range of the dentist, is it not a duty which we owe to the community to avail ourselves of every means which may be afforded, to extend our knowledge of medical science in general. Let us hope that the day is not far distant when the dentist no longer restricted to mere topical remedies shall be able to trace to their remote origin the causes of diseases which at this day are involved in obscurity save to the more enlightened discernment of the physician. A new era is dawning upon us, old things are passing away, worn out effete theories are being displaced by stern facts, while on the other hand every year as it passes brings back to our notice old discarded theories which present themselves, clothed in a new significance. As in the past we have surmounted formidable obstacles and disarmed prejudices no less formidable, so in the future we shall advance to a position of higher honor and greater dignity if we but avail ourselves of the opportunities which offer themselves as stepping-stones to our advancement. The medical profession will gladly reccive us on a common footing when we shall have qualified ourselves for the intelligent practice of medicine, and the public will more willingly award to us an adequate compensation for our services when it shall have come to be understood that we have a tangible claim to a standing above that of a mere artisan. Knowing, then, how best to subserve our own interests as a profession, let us neglect no opportunity to prepare ourselves for the more momentous responsibilities which are even now impending over us.

